# Mitochondrial DNA as a Biomarker for Acute Central Serous Chorioretinopathy: A Case-Control Study

**DOI:** 10.3389/fmed.2022.938600

**Published:** 2022-06-21

**Authors:** Noriyasu Hashida, Kazunobu Asao, Chikako Hara, Andrew J. Quantock, Ryotaro Saita, Hiroyuki Kurakami, Kazuichi Maruyama, Kohji Nishida

**Affiliations:** ^1^Department of Ophthalmology, Osaka University Graduate School of Medicine, Osaka, Japan; ^2^Structural Biophysics Group, School of Optometry and Vision Sciences, Cardiff University, Cardiff, United Kingdom; ^3^Department of Medical Innovation, Osaka University Hospital, Osaka, Japan; ^4^Department of Innovative Visual Science, Osaka University Graduate School of Medicine, Osaka, Japan; ^5^Integrated Frontier Research for Medical Science Division, Institute for Open and Transdisciplinary Research Initiatives (OTRI), Osaka University Graduate School of Medicine, Osaka, Japan

**Keywords:** mitochondrial DNA, high mobility group box 1 (HMGB1), 8-OHdG, central serous chorioretinopathy (CSCR), stress, biomarker

## Abstract

**Background:**

The literature suggests that stress may play a pivotal role in the precipitation of acute central serous chorioretinopathy (CSC) because chorioretinal integrity can be affected by the psychosocial state of the patient, indicating the need for a biomarker. Not only physical stress but also psychological stress causes many types of physical disorders. However, little is known about the pathophysiology of stress-induced disease. The objective of this study was to investigate whether serum factors might be involved in the development of stress-induced ocular diseases.

**Methods:**

This observational case series included 33 eyes of 33 consecutive patients with treatment-naïve acute CSC. Fifty eyes of 50 age-matched healthy volunteers were included in this study as non-CSC controls. Serum samples were collected from all participants, and the levels of mitochondrial DNA (mtDNA) were measured by quantitative real-time (RT)-PCR. Serum levels of high-mobility group box (HMGB) 1 and 8-hydroxy-2′-deoxyguanosine (8-OHdG), biological markers of acute/chronic inflammation and oxidative stress, were also measured. The relationships between serum mtDNA, 8-OHdG, and HMGB1 concentrations were investigated by multivariate regression analysis, alongside an assessment of clinical data.

**Results:**

In the treatment-naïve acute CSC group, the serum mtDNA levels (36.5 ± 32.4 ng/mL) were significantly higher than the levels in the control group (7.4 ± 5.9 ng/mL; *p* < 0.001). Serum levels of 8-OHdG and HMGB1 in treatment-naïve acute CSC patients measured 0.12 ± 0.08 ng/mL and 18.1 ± 35.0 ng/mL, respectively, indicating that HMGB1 levels were elevated in CSC compared with the control group. Multivariable regression analysis demonstrated that increased serum mtDNA levels were significantly associated with the height of serous retinal detachment.

**Conclusion:**

We showed serum mtDNA and HMGB1 level elevation and its relation to the clinical activities of CSC, indicating that serum mtDNA and HMGB1 could serve as biomarkers for the acute phase of the disease. The use of these biomarkers makes it possible to predict disease onset and determine disease severity.

## Introduction

Stressors that influence our mind and body include physical stressors (for example, heat, cold, noise and congestion), chemical stressors (pollutants, drugs, oxygen deficiency, carbon monoxide, etc.), and psychological/social stressors (human relations, job problems, family problems, etc.). Often, when we use the term “stress,” we are referring to psychological/social stressors, which can be divided into three main categories: psychological, physical, and behavioral. Various internal and external factors adversely affect the homeostasis of organisms from the molecular level to the whole-body level, causing stress. In response to such stress, a specific reaction corresponding to the type of stimulation and/or a series of non-specific biological stress reactions unrelated to the type of stimulation can be observed (1). These manifestations can include symptoms such as headache, stiff shoulders, back pain, eye fatigue and blurred vision, palpitations and shortness of breath, stomach ache, a loss of appetite, constipation, diarrhea, and insomnia. Various biomarkers have been traditionally reported to be useful in the diagnosis of diseases and monitoring after therapeutic intervention (2). A variety of physiological parameters have also been reported as stress biomarkers for psychological and physical stress states. Therefore, the detection and quantification of the degree of stress is very important for maintaining good health in the many stressful environments that people face today.

Central serous chorioretinopathy (CSC) is a chronic disorder that is most commonly seen in men between 30 and 60 years old and manifests as serous retinal detachment (SRD) in the macular area. This disease tends to spontaneously regress after several months but can recur frequently. CSC is reported to be a stress-induced and vision-threatening disease; it has been suggested that psychological stress may be partially involved in the pathophysiology of CSC. Various intrinsic and extrinsic factors have been reported to negatively and positively affect choroidal integrity and equilibrium, including predisposing factors such as sympathetic activity (3), a stress-prone personality (4), endogenous cortisol levels (5, 6), systemic corticosteroid use (7), pregnancy (8), and hypertension, tobacco intake, hypertension (9), all of which are reportedly involved in the pathogenesis of CSC (9, 10). Pathological examinations have shown the hyperpermeability of choroidal blood vessels and an increased choroidal thickness, resulting in SRD (11–13). Choroidal blood vessels experience the most abundant blood flow in the body, with innervated blood vessels contributing to the constriction and regulation of blood flow (14). Various studies have discussed the pathophysiology of CSC (9–15); however, it is not clear how blood vessel structure and/or blood flow are affected in the choroid of CSC patients.

Recently, endogenous mitochondrial DNA (mtDNA) was implicated in the pathogenesis of inflammatory disease (16). Generally, trauma leads to systemic inflammatory response syndrome (SIRS), with fatal outcomes. After severe injury, fragments of mtDNA are released into the circulation, where they cause severe systemic reactions. Indeed, recently, a relationship between plasma mtDNA levels and the severity of SIRS was shown to be correlative (17), and subsequent work also indicated that an increase in serum mtDNA concentration is related to chronic diseases with or without inflammation (18, 19). Numerous factors are reportedly involved in ocular disease development. For example, it has been reported that reduced serum levels of serotonin are present in chronic CSC patients (20), suggesting decreased serum serotonin levels as a potential biomarker for the chronic but not acute phase of the disease. Recently, circulating cell-free mitochondrial DNA has been reported to be involved in a variety of diseases (21, 22). Evidence that mitochondria are downstream targets of stress is provided by a range of studies showing that chronic stress affects the structure and function of mitochondria (21–23). In ocular diseases such as CSC, where stress may play a role in the pathogenesis of the disease, we hypothesized that chronic stress may affect the mitochondria, resulting in an increase in serum mtDNA, which may play a role in the pathogenesis of the disease. Psychological stress is also correlated with increased serum levels of mtDNA, which, as a damage-associated molecular pattern (DAMP), is a potent trigger of innate inflammatory responses (24). Among the variety of DAMPs, high-mobility group box (HMGB) 1, a prototypic member of the alarmin family and chromatin-binding nuclear DAMP, is also linked with inflammation and malignancies (25). Not surprisingly, mtDNA is intimately associated with oxidative insult to cells, resulting in the synthesis of reactive oxygen species such as 8-hydroxy-2′-deoxyguanosine (8-OHdG).

The premise of the current study is that changes in serum levels of mtDNA, HMGB1, and/or 8-OHdG might be involved in the onset or acute phase of CSC and that serum mtDNA levels in acute CSC might represent a stress-related disease biomarker.

## Materials and Methods

### Ethics Statement

All patients provided written informed consent for the investigation, and the study adhered to the tenets of the Declaration of Helsinki. This study was registered with the Osaka University Clinical Research Review Committee Registry (registration number, CRB5180007-13448). All participants provided written consent prior to blood collection.

### Patients

**FIGURE 1:**
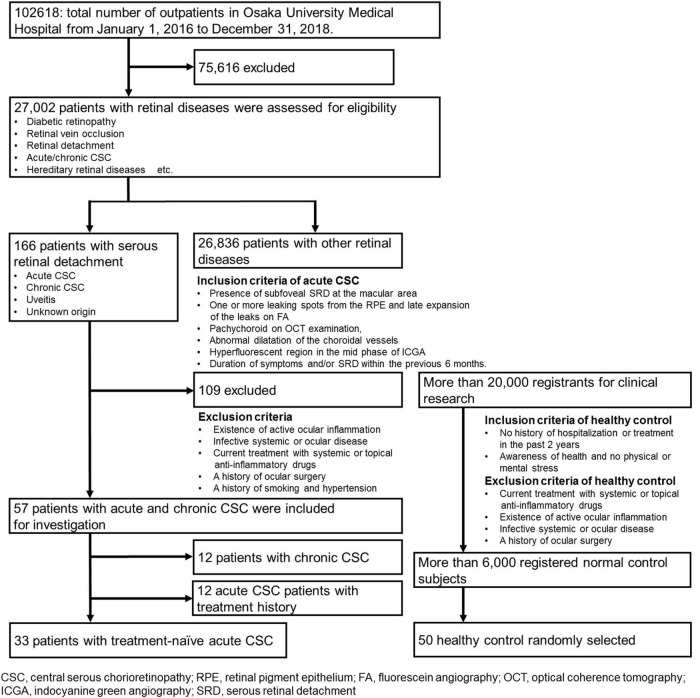
Flow diagram of patient enrollment and inclusion analysis.

Recruitment began on January 1, 2016, and ended on December 31, 2018. During this time, a total of 102,618 patients visited the patient portal, and 27,002 patients with retinal diseases were assessed for eligibility. A flow diagram of patient enrollment and inclusion analysis is shown in Figure 1. Finally, this observational case series included 33 eyes of 33 consecutive patients with treatment-naïve acute CSC (27 men, 6 women). Acute CSC was defined as CSC with a disease duration of symptoms and/or SRD within the previous 6 months. To ensure the accuracy of the acute onset and duration of the disease, the timing of the onset of the disease was verified by asking the patients directly and by referral letters from local ophthalmologists. Patient medical records were retrospectively reviewed, with a follow-up duration ranging from 3 to 45 months [16.6 ± 12.1 months (mean ± s.d.)]. Age-matched healthy subjects [*n* = 50; average age (mean ± s.d.) 49.8 ± 16.4 years] without ocular symptoms or systemic disease served as controls. The inclusion criteria and the acute CSC diagnostic criteria were as follows: the presence of subfoveal SRD in the macular area, one or more leaking spots in the retinal pigment epithelium (RPE) and the late expansion of the leaks on fluorescein angiography (FA), pachychoroid on optical coherence tomography (OCT) examination, and abnormal dilatation of the choroidal vessels and a hyperfluorescent region in the mid phase in indocyanine green angiography (ICGA). The timing of the onset of CSC had to be accurately estimated, and CSC patients were to be treatment-naïve. Exclusion criteria were the existence of active ocular inflammation; the presence of infective systemic or ocular disease; current treatment with systemic or topical anti-inflammatory drugs; hypertension and a history of smoking; a history of ocular surgery; and current retinal disease with the presence of an epiretinal membrane, diabetic retinopathy, retinal vein occlusion, and age-related macular degeneration. For the assessment of patients’ psychological stress, we applied the widely used Kessler 6 (K6) scale and checked the patients’ stress status (26). Demographic data such as observation periods, the height and basal area of subretinal fluids, choroidal thickness, visual acuity, and age were obtained and are shown in Supplementary Tables 1, 2. The data of patients with chronic CSC are shown in Supplementary Table 3 for comparison.

### Data Acquisition, Sample Collection and mtDNA Extraction

Retina specialists (NH, CH, and KM) diagnosed and classified the features of acute/chronic CSC. Peripheral blood was drawn, and ophthalmological examination was performed in the outpatient ward. All patients had all tests carried out systematically from their first visit once consent for participation in the study had been obtained. An outpatient optometrist acquired all imaging data. Blood samples were taken at the outpatient visit closest to the predicted onset of the disease. From all study participants (CSC patients and healthy controls), ethylenediaminetetraacetate (EDTA)-treated peripheral venous blood samples were collected after resting for 30 min at room temperature. The samples were centrifuged at 3,000 rpm for 15 min, after which separated serum was promptly frozen and stored at −80°C until the time of analysis. QIAamp DNA Mini Kits and Blood Mini Kits (Qiagen, Germantown, MD, United States) were used to extract mtDNA from the sera. Serum mtDNA levels were measured by NH and KA.

### Measurement of mtDNA by Real-Time PCR

Levels of mtDNA were measured by quantitative real-time (RT)-PCR using SsoAdvanced™ Universal^®^ SYBR Green Supermix (Bio-Rad laboratories, Inc., Japan) according to the manufacturer’s protocols. Primers for human cytochrome B (forward primer 5’-atgaccccaatacgcaaaat-3’ and reverse primer 5’-cgaagtttcatcatgcggag-3’) were synthesized by Invitrogen (Thermo Fisher Scientific, Co., Ltd., Japan). Primer sequences had no significant homology with the DNA found in any bacterial species searched by the Basic Local Alignment Search Tool (BLAST). RT–PCR standard curves were created to quantify the mtDNA concentration by using purified mtDNA and cytochrome B as targets. Samples that produced no PCR products after 40 cycles were considered “undetectable,” and the Ct was set to 40 for statistical purposes.

### Serum Levels of High-Mobility Group Box (HMGB) 1,8-Hydroxy-2′-Deoxyguanosine (8-OHdG/8-oxo-dG) and Cortisol

Serum HMGB1 and 8-OHdG levels were determined by ELISA at the Japan Institute for the Control of Aging, NIKKEN SEIL Co., Ltd., (Shizuoka, Japan). An experienced technician who was blinded to the sample details performed the assays. The reference range for serum 8-OHdG concentration was 0.1–0.3 ng/mL. Mean serum levels of HMGB1 in healthy controls have been reported to be 1.65 ± 0.04 ng/mL (25). Serum cortisol levels were measured with a Cortisol EIA Kit, DetectX, Strip Plate (96-well) (Arbor Assays Inc., Ann Arbor, MI, United States) according to the manufacturer’s protocol.

### Measurement of the Choroidal Thickness, Height of Serous Retinal Detachment, and Basal Area of Serous Retinal Detachment Using Swept Source Optical Coherence Tomography

To measure the choroidal thickness and height of SRD, we used swept source optical coherence tomography (SS-OCT) (DRI OCT-1; Topcon Corp., Tokyo, Japan) with a long-wavelength light source (1,060 nm). We manually measured the height of SRD in 6 × 6-mm images with densities of up to 256 (horizontal) × 256 (vertical) B-scan lines around the retina. The height of the SRD was defined as the vertical distance from the RPE to the bottom at the fovea. We also manually measured the foveal choroidal thickness (using software in the SS-OCT instrument) from the outer border of the RPE to the chorioscleral interface of the subfovea according to a previous report (27). The calculation procedure of the basal area of SRD in OCT images is shown in Supplementary Figure 2.

### Statistical Analysis

Statistical analysis was carried out using EZR (Saitama Medical Center, Jichi Medical University, Saitama, Japan), which is a graphical user interface for R (The R Foundation for Statistical Computing, Vienna, Austria) (28). The Mann–Whitney *U* test was used to compare factors between groups. The correlation between the log of serum mtDNA levels and other parameters (log of serum HMGB1 level and log of 8-OHdG level) was assessed using the Pearson correlation coefficient. A receiver operating characteristic (ROC) curve was used to determine the optimal cut-off value of serum mtDNA\ for predicting the risk of developing CSC. The ability of the serum mtDNA and HMGB1 levels to accurately predict the disease is presented by the area under the ROC curve (AUC). The ideal cut-off value corresponded to the maximal value of the Youden index. To investigate the relationship between the serum mtDNA concentration of treatment-naïve acute CSC patients and patient clinical data, we carried out univariable regression analyses, in which the serum mtDNA level was regressed on patient clinical variables (i.e., age, sex, visual acuity, total follow-up period, sampling times after onset, height of SRD, basal area of SRD, foveal choroidal thickness, and serum 8-OHdG and HMGB1 concentrations). Multivariable regression analysis was further performed to identify the factors most significantly associated with the serum mtDNA levels using the backward method based on a *p* value of 0.05. For all tests, *p* values less than 0.05 were considered significant.

## Results

### Elevation of Serum mtDNA Levels in Patients With Acute and Chronic Central Serous Chorioretinopathy

**FIGURE 2:**
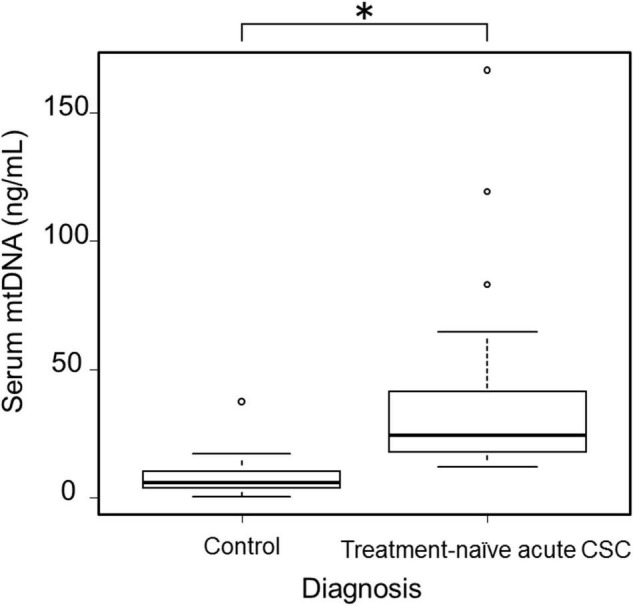
Boxplots showing serum mtDNA levels in healthy control participants (*n* = 50) and treatment-naïve acute CSC patients (*n* = 33). A significant increase in serum mtDNA levels was observed in treatment-naïve acute CSC patients (*p < 0.001). mtDNA, mitochondrial DNA; CSC, central serous chorioretinopathy.

Based on an application of our inclusion and exclusion criteria (see section “Materials and Methods”), a total of 33 patients with treatment-naïve acute CSC (from the initial group of 45 consecutive cases with acute CSC) and 50 healthy individuals were included in the study. With severe mental illness defined as a K6 score ≥ 13, we investigated the mental distress of the patients (26). Few patients had a K6 score > 13, but most patients had a 5 ≤ K6 score < 13, indicating that they were under moderate psychological distress. The demographic data of the 33 included treatment-naïve acute CSC patients are presented in Table 1. There were no significant differences in age, sex, or systemic conditions between the treatment-naïve acute CSC and healthy control groups. An SRD in the fovea was found in all cases. In the treatment-naïve acute CSC group, the serum mtDNA concentration [36.5 ± 32.4 ng/mL (mean ± s.d.); range, 12.0–166.8 ng/mL; median, 24.2 ng/mL; interquartile range, 17.7–41.4 ng/mL] was significantly higher than that in the control group [7.4 ± 5.9 ng/mL (mean ± s.d.); range, 0.4–37.5 ng/mL; median, 6.0 ng/mL; interquartile range, 3.7–10.2 ng/mL; *p* < 0.001] (Figure 2 and Table 2). In the majority (37 of 50 individuals) of the healthy control group subjects, serum mtDNA concentration levels did not exceed 10 ng/mL, whereas in the treatment-naïve acute CSC group, all 33 patients had levels in excess of 10 ng/mL. Even in the treatment-experienced acute CSC group, the serum mtDNA concentration [16.7 ± 7.4 ng/mL (mean ± s.d.)] was significantly higher than that in the control group (Supplementary Figure 1). The chronic CSC group included chronic and chronic recurrent CSC patients (Supplementary Table 3). In this group, the serum mtDNA concentration (7.0 ± 3.4 ng/mL (mean ± s.d.) was significantly lower than that in the treatment-naïve acute CSC group (Supplementary Figure 1).

**TABLE 1:** Demographic data of treatment-naïve acute CSC patients.

	Treatment-naïve acute CSC	Healthy control
No. eyes	33	.
Age, y, mean ± SD (range)	50.0 ± 10.5 (35–70)	49.8 ± 16.4 (22–77)
Sex, n (%)		
Male	27 (81.8)	41 (82.0)
Female	6 (18.2)	9 (18.0)
Total follow-up period, mo, mean ± SD (range)	16.6 ± 12.1 (3–45)	.
Sampling times after onset, mo, mean ± SD (range)	1.4 ± 0.7 (0.5–4)	.
No. of SRD, n (%)	33 (100)	.

*CSC, central serous chorioretinopathy; SRD, serous retinal detachment.*

**TABLE 2:** Clinical and labo data of treatment-naïve acute CSC patients and healthy.

	Treatment-naïve acute CSC	Healthy control
Maximum height of SRD, μm, mean ± SD (range)	268.9 ± 174.5 (82–730)	.
Basal area of SRD, mm^2^, mean ± SD (range)	11.5 ± 10.4 (1.07–49.28)	.
Choroidal thickness, μm, mean ± SD (range)	452.8 ± 109.4 (262–686)	.
Serum cortisol concentration, μg/mL, mean ± SD (range)	8.9 ± 4.0 (4.6–18.4)	7.8 ± 3.8 (3.3–13.5)
Serum mtDNA concentration, ng/mL, mean ± SD (range)	36.5 ± 32.4 (12.0–166.8)	7.4 ± 5.9 (0.4–37.5)
Serum 8-OHdG concentration, ng/mL, mean ± SD (range)	0.12 ± 0.08 (0.04–0.37)	0.10 ± 0.03 (0.06–0.22)
Serum HMGB1 concentration, ng/mL, mean ± SD (range)	18.1 ± 35.0 (2.4–148.3)	2.4 ± 1.8 (0.0–8.0)

*CSC, central serous chorioretinopathy; SRD, serous retinal detachment; mtDNA, mitochondrial DNA; 8-OHdG, 8-hydroxy-2′-deoxyguanosine; HMGB1, high mobility group box-1 protein.*

### Serum Levels of 8-Hydroxy-2′-Deoxyguanosine (8-OHdG), HMGB1, and Cortisol

**FIGURE 3:**
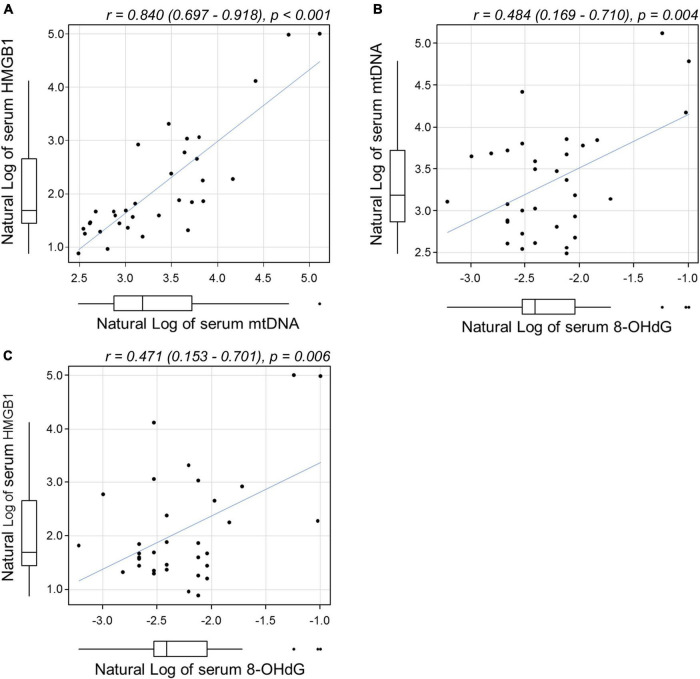
Scatterplots showing the relationship between **(A)** serum mtDNA and HMGB1, **(B)** mtDNA and 8-OHdG, and **(C)** HMGB1 and 8-OHdG levels by Pearson correlation analysis. All variables were natural log transformed. **(A)** A significant correlation was found between serum mtDNA and serum HMGB1 levels [*r* = 0.84 (0.697–0.918), *p* < 0.001]. Weak correlations were found between **(B)** mtDNA and 8-OHdG [*r* = 0.484 (0.169–0.971), *p* = 0.004] and between **(C)** HMGB1 and 8-OHdG [*r* = 0.471 (0.153–0.701), *p* = 0.006]. mtDNA, mitochondrial DNA; HMGB1, high-mobility group box (HMGB) 1; 8-OHdG, 8-hydroxy-2′-deoxyguanosine.

The concentration (mean ± s.d.) of 8-OHdG in the sera of patients with treatment-naïve acute CSC measured 0.12 ± 0.08 ng/mL, which, when compared with data in healthy controls (0.10 ± 0.03 ng/mL), indicated that values were within the normal range (29). On the other hand, the serum concentration of HMGB1 in treatment-naïve acute CSC patients was 18.1 ± 35.0 ng/mL (mean ± s.d.), which was higher than that in healthy controls (2.4 ± 1.8 ng/mL) (Table 2) (25). The serum concentration of cortisol in treatment-naïve acute CSC patients was 8.9 ± 4.0 μg/dL (mean ± s.d.), with no significant difference from the cortisol levels in healthy controls (7.8 ± 3.8 μg/dL) (Table 2). Pearson correlation coefficients were calculated to examine the correlation among serum mtDNA, HMGB1, and 8-OHdG levels (Figures 3A–C, all variables are natural log transformed). There was a weak correlation between serum mtDNA and serum 8-OHdG (*r* = 0.484, *p* = 0.004) and between serum HMGB1 and serum 8-OHdG (*r* = 0.471, *p* = 0.006) (Figures 3B,C). In particular, the results showed that the serum mtDNA and serum HMGB1 levels were significantly correlated (*r* = 0.840, *p* < 0.001) (Figure 3A).

### Evaluation of Potential Biomarkers of Central Serous Chorioretinopathy Development

**FIGURE 4:**
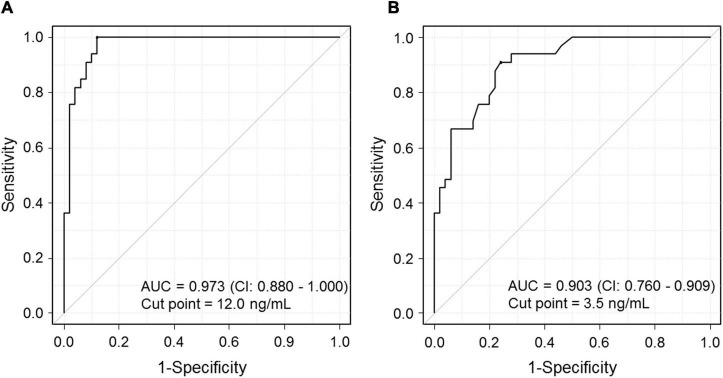
Graph showing the receiver operating characteristic (ROC) curve for predicting the risk of CSC development. **(A)** The ROC curve shows the relationship between serum mtDNA levels and the occurrence of CSC. Area under the ROC curve (AUC): 0.973. CSC, central serous chorioretinopathy. **(B)** The ROC curve shows the relationship between serum HMGB1 levels and the occurrence of CSC. AUC: 0.903. CSC, central serous chorioretinopathy; HMGB1, high-mobility group box (HMGB) 1.

To determine the sensitivity and specificity of serum mtDNA and HMGB1 levels for predicting CSC development, ROC curve analysis was used. The optimal cut-off value of the serum mtDNA and HMGB1 level with the highest sensitivity and specificity was 12.0 ng/mL and 3.5 ng/mL, respectively (Figures 4A,B). When the cut-off point for mtDNA was set at 12.0 ng/mL, 44 out of 50 (88%) of the healthy controls had mtDNA levels below this value, whereas in the treatment-naïve acute CSC group, all 33 patients had levels in excess of 12 ng/mL. Thus, an AUC of 0.973 (95% CI 0.942–1.000) was achieved with a sensitivity of 100% and specificity of 88.0% at the balanced operating point, showing outstanding discrimination (Figure 4A).

### Univariable and Multivariable Regression Analysis

Univariable regression analysis revealed that the height of SRD (*p* < 0.001), basal area of SRD (*p* = 0.024), serum HMGB1 levels (*p* < 0.001) and 8-OHdG levels (*p* < 0.001) were significantly associated with serum mtDNA levels (Table 3). Since the basal area of SRD was strongly correlated with the height of SRD, it was excluded from the multivariable regression analysis. Among these parameters, the height of SRD (*p* = 0.039) and serum HMGB1 levels (*p* < 0.001) were significantly associated with serum mtDNA levels in multivariable analysis. Of the clinical features assessed, elevated levels of serum mtDNA were found to be most significantly associated with the height of SRD (Table 3).

**TABLE 3:** Univariate and multivariate regression analysis of the association between serum mtDNA levels and clinical parameters in patients with treatment-naïve acute CSC.

Variable	Univariable linear regression			Multivariable linear regression *	
	Regression Coefficient	*R* ^2^	*P*-value	Regression Coefficient	*P*-value
Age	−0.955	0.095	0.081	.	.
Sex	9.481	0.013	0.526	.	.
Visual acuity	3.13	0.0013	0.839	.	.
Total follow-up period	−0.379	0.02	0.432	.	.
Sampling times after onset	−13.458	0.089	0.092	.	.
Height of SRD	0.103	0.306	<0.001	0.05	0.039
Basal area of SRD	1.225	0.154	0.024	.	.
Choroidal thickness	−0.031	0.011	0.559	.	.
Serum HMGB1 concentration	0.85	0.843	<0.001	0.78	<0.001
Serum 8-OHdG concentration	282.94	0.462	<0.001	.	.

*CSC, central serous chorioretinopathy; mtDNA, mitochondrial DNA; SRD, serous retinal detachment; HMGB1, high mobility group box-1 protein; 8-OHdG, 8-hydroxy-2′-deoxyguanosine.*

*Adjusted R^2^ = 0.855.

## Discussion

The main feature of CSC onset is the vascular hyperpermeability of the choroidal vasculature, which results in an increased foveal choroidal thickness (9, 12). The detailed mechanisms are not fully understood, but neurological or serum factors are presumed to be involved in the pathogenesis of CSC (9). Here, we indicate that serum mtDNA levels were significantly increased in patients with treatment-naïve acute CSC. Circulating HMGB1 levels were also elevated in the sera of treatment-naïve acute CSC patients. These results suggest that heightened levels of serum mtDNA and HMGB1 are likely risk factors for the occurrence of acute CSC. These factors might also be involved in the development of acute CSC, identifying them as potential biomarkers of the acute phase of CSC.

In the innate immune system, Toll-like receptors (TLRs) serve as sensors to recognize pathogen-associated molecular patterns (PAMPs) (30, 31) and DAMPs, which arise because of cellular injury (32). For example, trauma can cause premature cell death in SIRS, such as sepsis (33). Microbial PAMPs initiate the activation of innate immunity *via* TLRs (34), while mtDNA contains CpG DNA repeats and is recognized by TLR9, which leads to the activation of inflammatory and/or resident cells (32, 34, 35). Circulating cell-free mtDNA serves as an inflammatory factor in a number of diseases (19, 36) and here, we show that an elevation in serum mtDNA levels is a feature of CSC. Previous work has shown the gene and protein expression levels of TLR9 in CSC, especially in the endothelial cells of the choroidal vasculature, which is the primary locus of the disease (37). Collectively, these findings suggest the involvement of serum mtDNA and TLR9 in the pathophysiology of acute CSC.

The level of 8-OHdG, an oxidative stress marker of DNA damage, is usually elevated when systemic oxidative stress occurs (38). Given that mtDNA is involved in oxidation–reduction reactions and that its level is known to fluctuate due to changes in oxidation state (29), we expected that the 8-OHdG levels would be heightened in the sera of acute CSC patients. Nevertheless, 8OHdG concentrations were within the normal range and did not show a statistically significant increase. Furthermore, mtDNA and 8-OHdG concentrations showed a weak correlation in the correlation analysis, but no significant correlation in the multivariable regression analysis. That they were not seems to suggest that oxidative stress is not centrally involved in CSC pathophysiology. In previous reports, endogenous cortisol was also found to be associated with the development of acute CSC, and it was proposed that serum corticosteroids might be related to the initiation of CSC development (6). We found no significant increase in serum cortisol levels in our cases, suggesting that serum cortisol might initiate CSC but that it does not reflect disease activity in the early phase of the disease.

In contrast to the unchanged concentrations of 8-OHdG and cortisol in the sera of acute CSC patients studied here, serum HMGB1 levels were significantly elevated. HMGB1, an alarmin molecule, is reportedly a biological marker of inflammation and malignancies (25, 39). HMGB1 is implicated in many types of disease and has many intracellular and/or extracellular functions. The extracellular functions of HMGB1 are mediated by its receptors, which include TLRs (40), which are activated by DNA-containing immune complexes with HMGB1 in response to nucleic acid release. Previous investigators have suggested that a sympathetic-parasympathetic imbalance causes CSC (41). Another report described how acute psychological stress increases cell-free mtDNA levels circulating in the serum (23, 24). The interaction between mtDNA and HMGB1 also reportedly plays an important role in tumor growth *via* TLR9 signaling (42). These findings suggest that physical and/or psychological stress might induce a sympathetic-parasympathetic imbalance and lead to an increase in serum mtDNA and HMGB1 levels, resulting in increased permeability of the choroidal vasculature through TLR9 signaling.

Lotery A et al. reported that ten per 100,000 men and two per 100,000 women in the population develop CSC each year (43, 44). In light of these reports, the 57 cases analyzed in this study are a reasonable number of new cases; however, further studies with larger cohorts are required to validate our findings. ROC curve analysis allowed us to calculate the cut-off point. In the chronic CSC group, only one case was above the cut-off value, suggesting that the optimal cut-off value may help detect acute CSC, differentiate chronic CSC from acute CSC, and predict recurrence.

The question arises as to whether elevated serum mtDNA or HMGB1 levels are a cause or a consequence of the disease. We conclude that elevated serum mtDNA or HMGB1 levels are a cause of CSC because previous investigators reported that intraperitoneal injection of mtDNA triggered inflammation in an *in vivo* experimental model (45), and our *in vitro* cell culture experiments showed that there was no increase in cytokines in CD14 + monocytes, but there was an increase in vascular endothelial growth factor (VEGF) and platelet-derived growth factor (PDGF)-AA in an RPE cell culture after the cells were exposed to CSC patient serum (Supplementary Figures 3, 4). If the elevation of serum mtDNA is a consequence of the disease, all patients in the acute phase of the disease should have elevated serum mtDNA, regardless of whether the disease is initial-onset or recurrent. The absence of elevated serum mtDNA in all patients with recurrent CSC strongly suggests that it may be a cause of the disease. A future comprehensive analysis, including factors not analyzed in the present study, will be necessary.

The main limitation of our study was the small sample size. However, the recruitment period of 3 years was sufficient to obtain sufficient clinically and biochemically complete patient data to evaluate the pathogenesis of CSC. The current study focused on the clinical findings only in the eye and analyzed the association with biomarkers but did not provide detailed information on pre-existing systemic conditions such as hypertension, a history of smoking and medication, employment status or mental health. Regarding the mental health, the K6 score did not show a predominant correlation with the clinical features such as SRD height, SRD area, serum levels of mtDNA and HMGB1 (data not shown). This is because the feeling of stress as assessed by the K6 score is subjective; therefore, the objective stress biomarker in this study may be useful in detecting stress conditions in the eye. A more comprehensive search for associations between systemic conditions and biomarkers is needed in the future.

In summary, we present data from a retrospectively assessed cohort of 33 patients that offer some potential novel insights into the pathophysiology of acute CSC. We also provide good evidence that psychological stress significantly induces ocular disease that might lead to severe visual impairment through biological processes. Further studies are required to ascertain whether the documented increases in serum mtDNA and HMGB1 levels are primary or secondary events in the development of acute CSC, although the elevated levels do identify mtDNA and HMGB1 as potential new biomarkers for acute CSC.

## Data Availability Statement

The original contributions presented in this study are included in the article Supplementary Material, further inquiries can be directed to the corresponding author/s.

## Ethics Statement

The studies involving human participants were reviewed and approved by Osaka University Clinical Research Review Committee Registry (registration number, CRB5180007-13448). The patients/participants provided their written informed consent to participate in this study.

## Author Contributions

NH and KN designed the study. NH, KA, CH, and KM gathered the data. NH, RS, and HK performed statistical analysis. RS and HK were statisticians who performed the statistical validity analysis. NH, AQ, and KN analyzed the data and vouch for the data and the analyses. NH was responsible for the decision to submit the manuscript for publication. All authors contributed to the writing of the manuscript, reviewed the manuscript, and amended or approved the final version.

## Conflict of Interest

The authors declare that the research was conducted in the absence of any commercial or financial relationships that could be construed as a potential conflict of interest.

## Publisher’s Note

All claims expressed in this article are solely those of the authors and do not necessarily represent those of their affiliated organizations, or those of the publisher, the editors and the reviewers. Any product that may be evaluated in this article, or claim that may be made by its manufacturer, is not guaranteed or endorsed by the publisher.

## Abbreviations

CSC: central serous chorioretinopathy
mtDNA: mitochondrial DNA
HMGB1: high-mobility group box 1
8-OHdG: 8-hydroxy-2′-deoxyguanosine
SRD: serous retinal detachment
DAMP: damage-associated molecular pattern
RPE: retinal pigment epithelium
FA: fluorescein angiography
OCT: optical coherence tomography
ICGA: indocyanine green angiography
ROC: receiver operating characteristic
AUC: area under the curve
TLRs: Toll-like receptors
PAMPs: pathogen-associated molecular patterns
SIRS: systemic inflammatory response syndrome
VEGF: vascular endothelial growth factor
PDGF: platelet-derived growth factor.
