# Supplementary material for: Mitochondrial DNA as a Biomarker for Acute Central Serous Chorioretinopathy: A Case-Control Study
Source: Front Med (Lausanne). 2022 Jun 21;9:938600. doi: 10.3389/fmed.2022.938600 (PMC9253465; doi:10.3389/fmed.2022.938600)
Supplement: Supplementary file 1 [file Data_Sheet_1.docx]

Supplementary Material

# Supplementary Figures

**
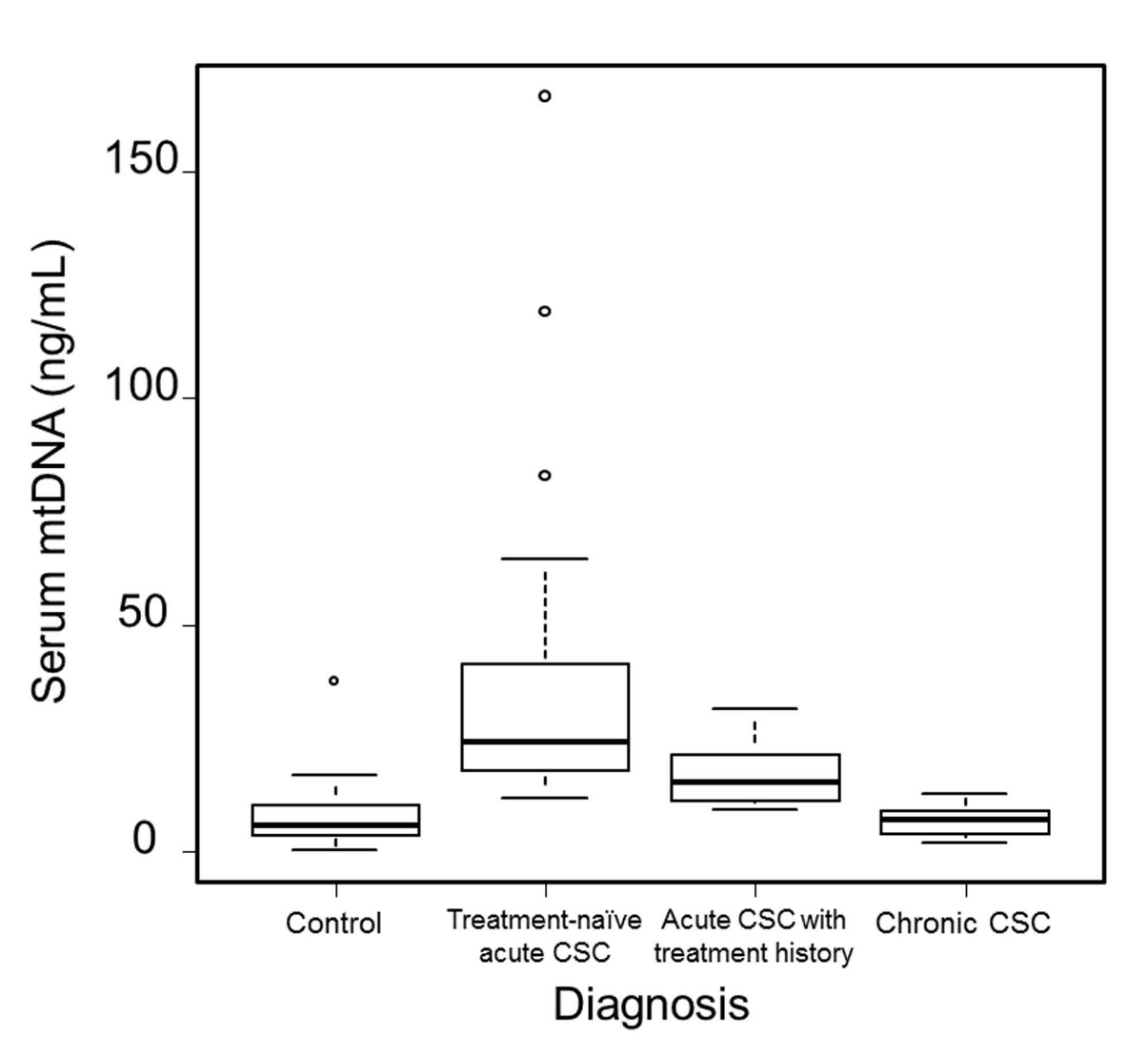
**

**Supplementary Figure 1. Serum mtDNA levels in in patients with CSC**

Boxplots showing serum mtDNA levels in healthy control participants (n=50), treatment-naïve acute CSC patients (n=33), acute CSC patients with a treatment history (n=12), and chronic CSC patients (n=12). The Kruskal–Wallis test was used to compare factors among groups, followed by the Mann–Whitney U test with Bonferroni correction. Control vs treatment-naïve acute CSC: *p <* 0.001; control vs acute CSC with treatment history: *p <* 0.001; control vs chronic CSC: *p* *=* 1.000; treatment-naïve acute CSC vs acute CSC with treatment history: *p* *=* 0.028; treatment-naïve acute CSC vs chronic CSC: *p <* 0.001; acute CSC with treatment history vs chronic CSC: *p* *=* 0.001. A significant increase in serum mtDNA levels was observed in treatment-naïve acute CSC patients (*p <* 0.001) and treatment-experienced acute CSC patients (*p <* 0.001). mtDNA, mitochondrial DNA; CSC, central serous chorioretinopathy.


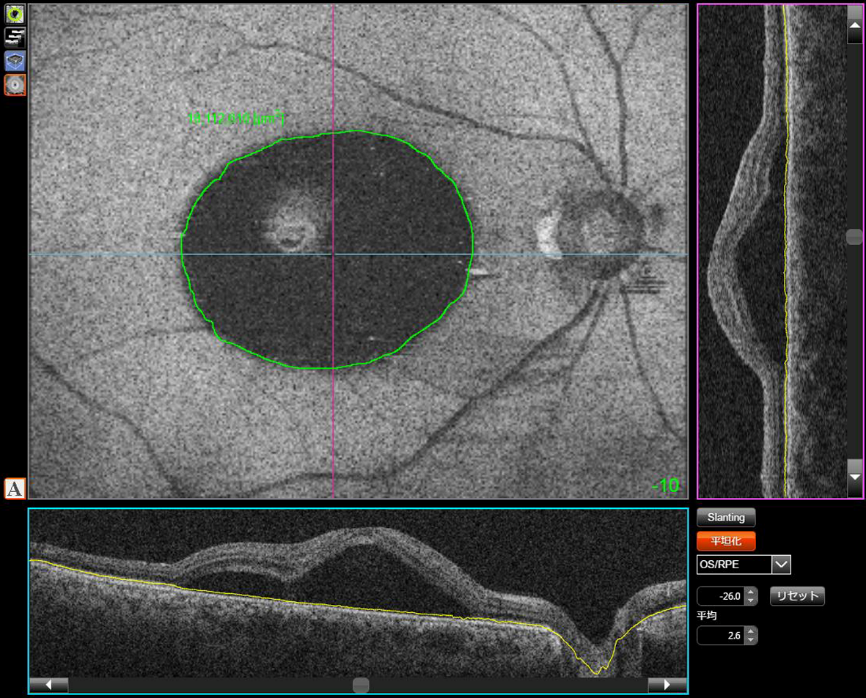


**Supplementary Figure 2. Detection and measurement of the basal area of subretinal detachment (SRD).**

After acquiring consecutive en face OCT images of SRD, the photoreceptor outer segment (OS)/retinal pigment epithelium (RPE) interface was automatically detected, and the RPE line was planarized by the software supplied with the machine. Using this image, the basal surface of the SRD was manually traced (green line), and the basal area was calculated.

**
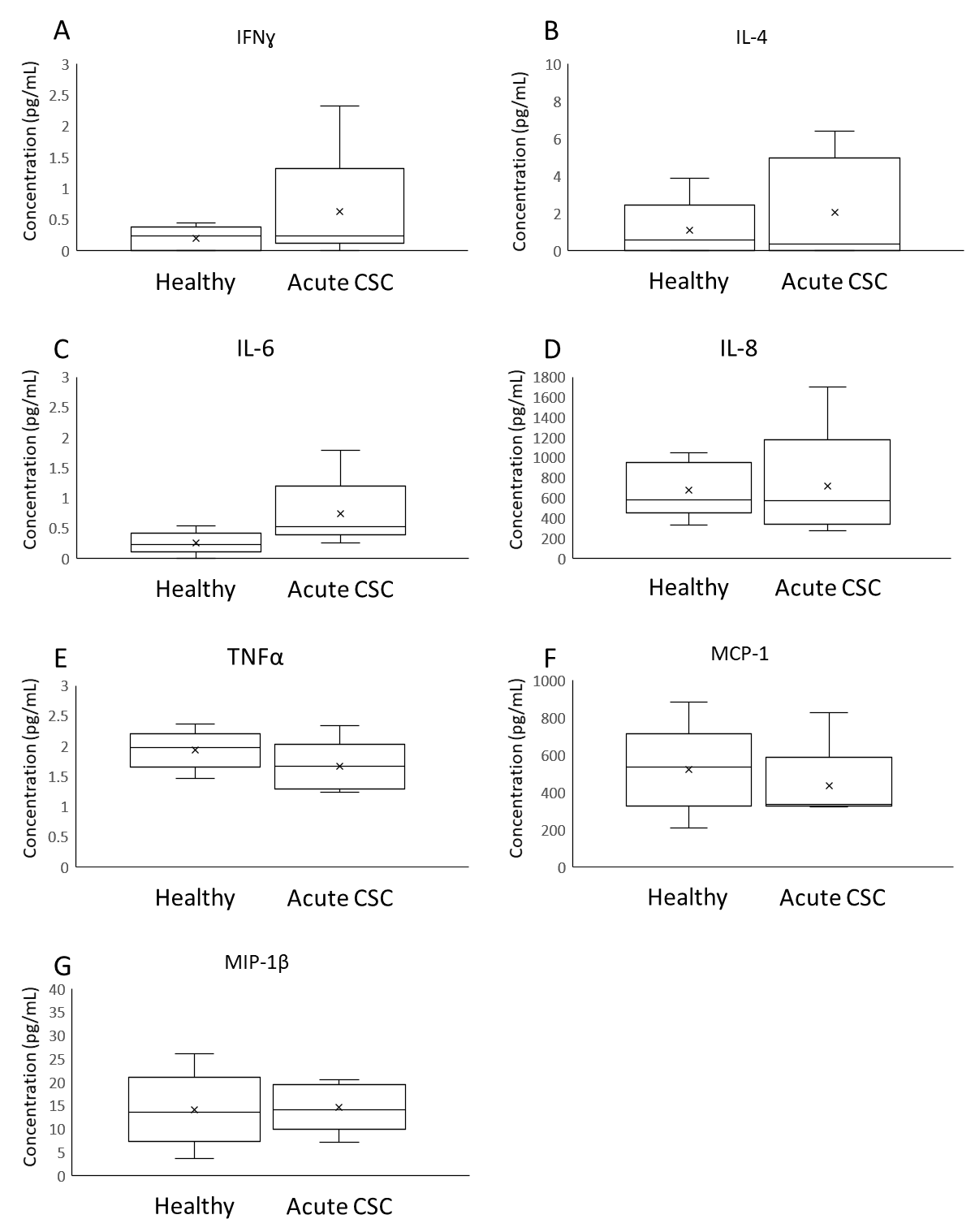
**

**Supplementary Figure 3. Comparison of cytokine levels in the culture supernatant of CD14^+^ monocytes**

Concentrations of (A) interferon-gamma (IFNγ): *p* = 0.74; (B) interleukin (IL)-4: *p* = 0.99; (C) IL-6: *p* = 0.09; (D) IL-8: *p* = 0.84; (E) tumour necrosis factor (TNFα) *p* = 0.22; (F) monocyte chemoattractant protein-1 (MCP-1): *p* = 0.42; and (G) macrophage inflammatory protein-1β (MIP-1β): *p* = 0.84 are shown according to the Mann–Whitney U test.

**
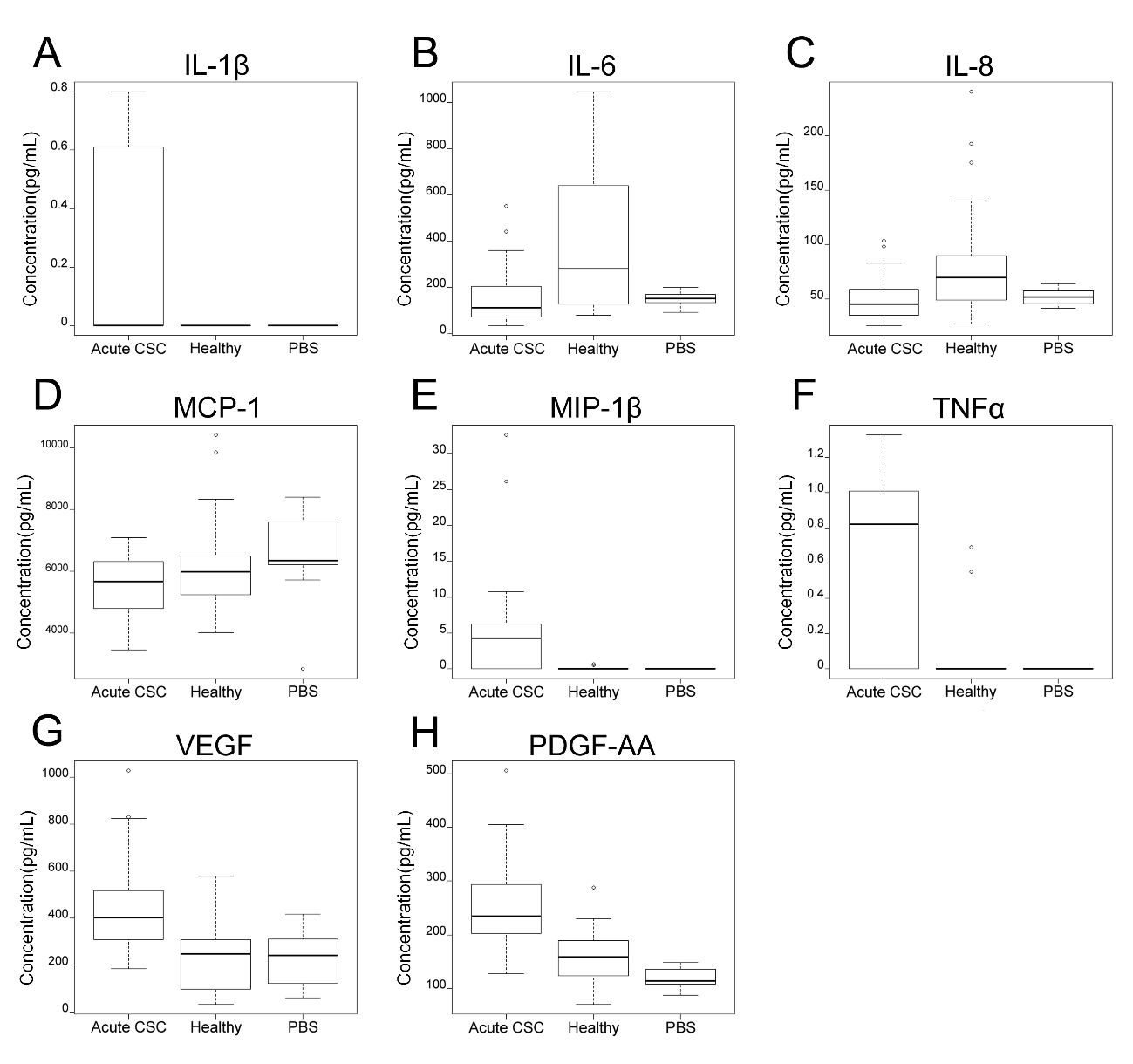
**

**Supplementary Figure 4. Comparison of cytokine levels in the culture supernatant of human RPE cells**

Concentrations of (A) interleukin (IL)-1β: *p* < 0.001; (B) IL-6: *p* < 0.001; (C) IL-8: *p* = 0.01; (D) monocyte chemoattractant protein-1 (MCP-1): *p* = 0.002; (E) macrophage inflammatory protein-1β (MIP-1β): *p* < 0.001; (F) tumour necrosis factor (TNFα): *p* < 0.001; (G) vascular endothelial growth factor (VEGF): *p* < 0.001; and (H) platelet-derived growth factor (PDGF) - AA: *p* < 0.001 are shown according to the Kruskal–Wallis test. RPE: retinal pigment epithelium, PBS: phosphate buffered saline.

**Supplementary Tables**

**Supplementary Table 1. Demographic data of treatment-naïve acute CSC patients**

**Supplementary Table 2. Demographic data of acute CSC and treatment history**

**Supplementary Table 3. Demographic data of chronic CSC patients**

**Supplemental Materials and Methods**

***Cell culture***

For the culture of human CD14^+^ monocytes, with reference to a previously published study, mononuclear cells were isolated by Ficoll/Paque density gradient centrifugation from buffy coats obtained from healthy donors. A subset of experiments was performed by means of positive selection of CD14-labelled target cells using a human magnetic antibody cell sorting (MACS) system (Miltenyi Biotec, Bergisch-Gladbach, Germany) according to the manufacturer´s protocol. Micromagnetic-selected CD14^+^ cells were cultured in RPMI-1640 culture medium (Sigma Immunochemicals, USA) supplemented with 10% foetal bovine serum (FBS) at 6 × 10^4^ cells/well in 96-well plates. Regarding the culture of human retinal pigment epithelial (RPE) cells, after the preincubation of human retinal pigment epithelial cells (HRPEpiC (6540, ScienCell) and HRPE (194987, Lonza) with Epithelial Cell Medium (EpiCM) (ScienCell), cells were subcultured on a Transwell (Corning) insert coated with 40 μg/mL mouse laminin (Thermo) and poly-L-lysine (Sigma) at 5 × 10^4^ cells/well in a 96-well plate. After culturing in EpiCM for 4 days to reach confluence, polar differentiation was induced in RPE medium. After differentiation, further culturing using Dulbecco’s modified Eagle medium (DMEM; Thermo) supplemented with 3% FBS and insulin-transferrin-selenium (ITS) media supplement (Thermo) was performed.

***Cytokine measurements***

After confirming cell confluency, 5 µL of patient serum was added to the culture medium, and the cells were incubated. The culture supernatant was collected 24 hours after incubation, and the samples were analysed using a Human Cytokine/Chemokine Magnetic Bead Panel (Merck Millipore, Darmstadt, Germany) according to the manufacturers’ instructions. This multiplex immunoassay contains premixed fluorogenic beads with monoclonal antibodies for the detection of **interferon-gamma (IFNγ), interleukin (IL)-1β, IL-4, IL-6, IL-8,** monocyte chemoattractant protein-1 (MCP-1), macrophage inflammatory protein (MIP)-1**β,** tumour necrosis factor-alpha (TNFα), vascular endothelial growth factor (VEGF), and platelet-derived growth factor (PDGF)-AA.
